# Bimodal distribution of trauma-related acute kidney injury (TrAKI): A clinical review

**DOI:** 10.2478/jccm-2026-0009

**Published:** 2026-01-30

**Authors:** Eleni Sertaridou, Christina Alexopoulou, Vasilios Papaioannou

**Affiliations:** ICU Department, University Hospital of Alexandroupolis, Alexandroupolis, Greece; Medical School, Democritus University of Trace; ICU Department, University Hospital of Alexandroupolis, Alexandroupolis, Greece

**Keywords:** trauma-related AKI, post-traumatic AKI, risk factors, pathophysiology

## Abstract

Severe trauma remains the leading cause of mortality and disability among young adults. Trauma-related Acute Kidney Injury (TrAKI) has been associated with worse outcomes, increased healthcare costs, and higher morbidity among survivors. The review aims to evaluate, from a pathophysiological perspective, the risk factors for TrAKI at different time points of trauma treatment, highlighting the need for early diagnosis of the syndrome and the implementation of preventive measures.

TrAKI is triggered at the time the injury occurs and further worsened by factors related to resuscitation process and potential complications. Severe trauma, due to hemorrhagic shock, is considered to act as the first hit. All subsequent necessary lifesaving procedures applied in trauma management, such as fluid resuscitation, massive transfusion and emergency surgery, could act as second hit, triggering “early” TrAKI, within 24–72 hours, due to renal hypoperfusion, hypoxia and reperfusion injury (R/I). The following critical care treatment, seems to act as the final third hit, resulting in “late” TrAKI appeared in 5–7 days or even later, caused by distal complications.

The incidence of TrAKI shows a biphasic pattern, with an “early “peak within 2–3 days after trauma, and a “delayed” occurring a week or later. This distinction could be of clinical importance because of its disparate pathophysiology and outcome. Early recognition of risk factors and diagnosis of TrAKI could improve the application of preventive measures and therapeutic treatment, reducing its prevalence.

## Introduction

Major trauma remains a considerable reason of admissions in surgical departments and Intensive Care Units (ICUs), and the principal cause of mortality and disability among young adults [1,2,3,4]. However, prehospital advanced life support, damage control surgery and evidence-based protocols for acute care of injuries have improved the trauma-related survival throughout the so-called “Golden hour”, while the progress in critical care medicine have suppressed its early mortality [3,5,6]. Consequently, an increasing number of delayed complications occur weeks or months later among polytrauma survivors of early effects of traumatic injury, due to severe critical illness and prolonged ICU and hospital stay [3,4,7].

The onset of Trauma-related Acute Kidney Injury (TrAKI) emerges as an independent risk factor for an almost threefold raise in mortality [3], extending ICU and hospital stay and enlarging healthcare costs [8], while the survivors are at substantial risk of chronic kidney disease (CKD) [9]. In contrast to medical illnesses Acute Kidney Injury (AKI), post-traumatic renal impairment has a clearly defined time of insult, making it an attractive target for potential prevention interventions [3]. Trauma patients experience specific exposures to a combination of AKI risk factors in different time points of trauma treatment, such as hemorrhagic and hypovolemic shock, aggressive resuscitation with massive transfusion and fluid infusion, systemic inflammation, rhabdomyolysis (RM), abdominal compartment syndrome (ACS) and major and/or multiple surgeries [3].

**Fig. 1.**
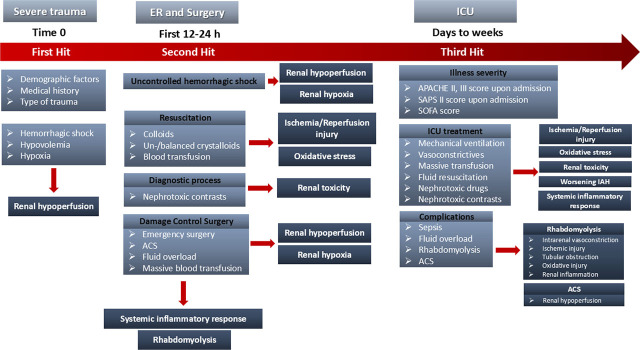
**Potential risk factors of TrAKI in different timepoints of trauma treatment: Severe trauma triggers initial AKI due to hemorrhage, hypovolemia and hypoxia, resulting in renal hypoperfusion. After these, the second hit due to fluid resuscitation, massive transfusion, emergency abdominal surgery, abdominal compartment syndrome and diagnostic processes with nephrotoxic contrast agents could further deteriorate renal hypoperfusion, cause ischemia/reperfusion injury, oxidative stress and renal toxicity. The third hit, due to critical care treatment or late complications, may cause additional disorders resulting in renal function impairment.** ACS: Abdominal Compartment Syndrome, APACHE score: Acute Physiology and Chronic Health Evaluation score, ER: Emergency Room, IAH: Intrabdominal Hypertension, ICU: Intensive Care Unit, SAPS score: Simplified Acute Physiology Score, SOFA score: Sequential Organ Failure Assessment score, TrAKI: Trauma related Acute Kidney Injury

In particular, severe trauma constitutes the first hit, triggering initial AKI due to hemorrhage, hypovolemia, hypotension, renal hypoperfusion and hypoxia. After these, the continued hemorrhage, the resuscitation process, including aggressive fluid infusion, massive transfusion and major abdominal or orthopedic surgical interventions, and their direct consequences, such as intrabdominal hypertension (IAH), ACS, RM and post-traumatic inflammatory response, seem to act as the second hit. The following prolonged critical care treatment, that includes mechanical ventilation support and need for vasoconstrictive drugs and possible nephrotoxic antibiotic agents, and its potential complications, such as RM, ACS and sepsis, could act as the third hit, deteriorating the fragile renal homeostasis (Figure 1).

**Fig. 2.**
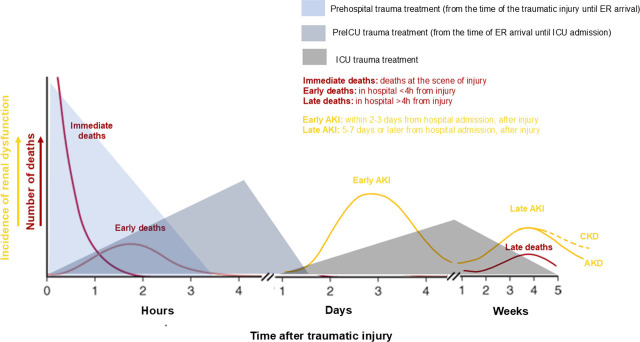
Bimodal distribution of Trauma – related Acute Kidney Injury (TrAKI): In line with trimodal distribution of deaths in trauma (red lines) [3], a bimodal distribution of TrAKI is observed (yellow lines). A first peak occurs approximately at 48–72 hours after trauma (time 0), is referred as “Early AKI” and is a direct consequence of trauma itself as well as prehospital and upon admission in the hospital trauma management. A second peak occurs >7 days or week, is referred as “Late AKI” and it is related to critical care and the delayed complications of critical illness. The latter AKI may exist as long as 90 days, hence called AKD (Acute Kidney Disease) (continuous yellow line) or rarely even longer, hence called CKD (chronic kidney disease) (dashed yellow line).

Consequently, TrAKI emerges a biphasic distribution, since a first peak of “early” AKI appears within 24–72 hours, related to trauma itself and instant life-saving interventions at trauma scene, during transfer and upon emergency (ER) or operation room (OR) treatment [10]. A second peak of “late” AKI occurred in 5–7 days or even later due to distal complications of trauma or intensive treatment and critical illness [11] (Figure 2). This discrimination could be crucial, as it seems to reflect individual pathophysiology and unique prognosis [12]. Enlarge upon individual pathophysiology, recognize various risk factors for TrAKI, that emerge throughout the management of trauma, highlighting proper early TrAKI diagnostic biomarkers and feature targeted prevention measures [13]. Moreover, acknowledge the unique prognosis, since “early” TrAKI usually has complete renal recovery [14,15,16], while “late” TrAKI more often develop CKD, reflects different possible outcome [10] and economic encumbrance, since prolonged renal replacement therapies (RRTs) increase distal morbidity and mortality and enlarge health-care costs [17].

The primary objective of this review is to evaluate the etiopathophysiologic risk factors of TrAKI in different time points of trauma treatment, identifying polytrauma patients, likely to develop AKI. Secondarily, this could highlight potential diagnostic biomarkers of AKI and preventive interventions capable to intercept worsening of the syndrome.

## Epidemiology of Trauma – Induced AKI

There is wide variability in the published incidence rates of TrAKI, ranging from 0.54% [9] to 37% [11] (Table 1). This variation is partly attributed to the different terminology used, such as “renal insufficiency”, “renal dysfunction”, or “acute renal failure”, all of which in latest publications have been substituted to the term “AKI” [9]. Moreover, the disparity in time of AKI diagnosis [10,16,18], the applied diagnostic criteria [19,20,21], and the known limitations of AKI definition, impinge on prevalence [22]. Besides, this discrepancy can be explained by the heterogeneity of studied populations. Cohorts that included only ICU-trauma patients reported higher prevalence of TrAKI [9,12,23], while others that contained more specific population, like traumatic brain injury patients [14,15,18], or only survivors trauma patients [15] presented lower incidences.

**Table 1.** Trauma-related AKI studies

	**Type of study**	**Material**	**AKI incidence**	**Diagnostic criteria**	**Time to AKI diagnosis**	**RRT requirement**	**Total mortality**	**Demographic/Comorbidities**	**Prehospital risk factors**	**Pre-ICU risk factors**	**ICU risk factors**	**Renal outcome**
Bagshaw SM et al, Ren Fail.2008;30:581–9 (ANZICS and APD). [12]	Multi-center retrospective study (57 ICUs) (1/1/2000 – 31/12/2005)	ICU only trauma patients (42.2% TBI)	1711/9449 (18.1%)	RIFLE	Within 24 hours after ICU admission in 36.1%	Not mentioned	16.7% AKI vs 7.8% non-AKI	Older age, Female sex, Pre-existed comorbidities	Direct renal injury, Abdominal and pelvic injury	Need for nephrectomy	APACHE II and III score, Sepsis	Not mentioned
Moore AM et al, Ren Fail. 2010;32(9):1060–1065.[18]	2-center retrospective study (Trauma DataBase) (1/1/2008 – 31/12/2008)	ICU only head trauma patients (GCS<13)	19/207 (9.2%)	RIFLE	First 10 days of admission	Not mentioned	42.1% AKI vs 18.1% non-AKI	Older age	Lower GCS, APACHE III score	Not mentioned	Not mentioned	Not mentioned
Bihorac A et al, Ann Surg. 2010;252(1):158–165.[10]	Multicenter prospective cohort (Trauma-DataBase) (11/2003-3/2008)	Trauma patients that live >24 h after injury	253/982 (26%)	RIFLE	First 28 days of hospitalization, 68% within 48 hours	11% RRT	3 times higher in AKI patients	Not the age	Low body temperature, Not ISS	Lactate level, blood transfusion	MOD score >3	50% didn’t have complete recovery in the first 28 days
Li N et al, Neurocrit Care. 2011;14(3):377–381.[14]	Retrospective single center study (1/2007 – 5/2010)	Only traumatic brain injury patients (GCS<8) with hospital stay>48 h	31/136 (23%), 21/31 (68%) stage 1, 7/31 (22%) stage 2, 3/31 (10%) stage 3	AKIN	Within 7 days after brain injury	0% RRT	17/31 AKI (55%) vs 11/105 non-AKI (11%)	Age	Lower GCS, Higher TBI score	Transtentorial herniation	Not mentioned	100% renal recovery among survivors
Shashaty MGS et al, J Crit Care 2012;27(5):496–504. [11]	Single center prospective cohort study (10/2005 – 6/2009) Excluded only head trauma	ICU trauma patients	147/400 (36.8%)	AKIN	First 5 days from ER presentation, 53.1% on day 0–1	9/147	1: 9.8% 2: 13.7% 3: 30.4% vs 3.8% in non-AKI	African American race, BMI>30, Diabetes	AIS>4	Blood transfusion	ISS	Not mentioned
Podoll AS et al, PLoS One. 2013; 8(10):e7737. [1]	Retrospective observational single center study (trauma database) (1/2009 – 3/2010) Excluded head trauma and burns	ICU trauma patients in Texas Trauma Institute	54/901 (6%), Stage 1: 85% 2: 11% 3: 4%	AKIN	Within 72 hours of admission	10/54 (19%) RRT	83/901 (9.2%) total mortality vs 16/54 (29.6%) AKI patients’ mortality	Age	AIS, Not ISS	Not mentioned	Not mentioned	Not mentioned
Baitello AL et al, J Bras Nefrol. 2013; 35(2):127–131. [24]	Retrospective observational study (7-8/2004)	Severe trauma patients (ISS>16) admitted in hospital	13/75 (17.3%)	AKIN	Within the first 3 days of admission	1/13 (7.6%) RRT	29.3% total mortality, 8/13 AKI mortality	Not age, Not gender	Head injury (GCS<10), ISS	Higher volume replacement, Not MAP	Not nephrotoxic drugs	Not mentioned
Skinner DL et al, Injury 2014;45(1):259–64. [25]	Retrospective observational single center (3/2008 – 3/2011)	ICU trauma patients	102/666 (15%): 25% (25/102) I, 57% (58/102) F	RIFLE criteria	57% at the time of ICU admission	39/102 (38%) RRT	57% AKI group, 78.9% among renal injury	Age>50,	ISS>45	BE >-12, iv contrast administration, blunt trauma	SOFA, RM	Not mentioned
Ahmed M et al, Br J Neurosurg. 2015;29(4):544–548.[15]	Retrospective observational study (1/4/2012 - 31/3/2013)	Only TBI patients, that underwent surgery, survived and hospital discharged	11/95 (11.6%): Stage 1: 7/11 (63.6%), 2: 3/11 (27.3%), 3: 1/11 (9.1%)	AKIN	81.8% within 5 days of admission	0/11 RRT	No mortality (0%)	Not significantly different	Lower GCS	Higher glucose, Larger volume of blood loss	Aminoglycoside therapy	100% renal recovery
Elterman J et al, J Trauma Acute Surg 2015;79(4 Suppl 2):S171–4. [26]	Retrospective cohort study (01/2010 – 11/2010)	Only trauma US army, with CPK>5000 U/L	79/318 (24.8%) with CPK>5000 U/L, AKI Stage I: 56/318 (17.6%) Stage 2: 3/318 (0.9%), Stage 3: 7/318 (2.2%)	KDIGO 2012	Not mentioned	6/7 Stage 3 required RRT	Not mentioned	Not mentioned	ISS, Mechanism of injury, Transport time	Massive transfusion >10 PRBC	Not mentioned	Not mentioned
Eriksson M et al, J Trauma Acute Care Surg. 2015;79(3):407–12. [16]	Single-center retrospective observational study (2/2007 – 9/2012)	Only ICU trauma patients	101/413 (24.9%) KDIGO stage 1: 59% 2: 13% 3: 28%	KDIGO 2012	2–7 days of ICU admission	27/101 (26%) CRRT, 6/101 IRRT	26.2% vs 7.1%	Male sex, Age, Diabetes	ISS score >40	Massive transfusion, HES overload	Shock, Sepsis,	None of ICU survivors were dialysis dependent 1 year after trauma
Heegard KD et al, J Trauma Acute Care Surg 2015;78(5):988–93. [23]	Data from 2 observational single-center studies	Only trauma ICU patients (mainly males) (Afghanistan – US army)	46/134 (34.3%) KDIGO stage 1: 25.4% 2: 3.7% 3: 5.2% (total: 34.3%)	KDIGO 2012	80.5% the first 2 hospital days	Not mentioned	AKI 21.7% vs non-AKI 2.3%	Not the age	ISS score	Lactate, Blood transfusion	Blood transfusion	Not mentioned
Stewart IJ et al. Am J Kidney Dis. 2016;68(4):564–570. [27]	Retrospective observational study (2/2002 – 2/2011)	Only ICU trauma US service members, wounded in Iraq or Afghanistan, transferred in Landstuhl, Germany	474/3807 (12.5%), Stage 1: 9.8%, 2: 1.6%, 3: 1.1%	KDIGO 2012	Within 7 days	14/474 RRT	112/3807 (2.9%) in total died, 13.1% with AKI vs 1.5% without AKI	Age, African American race	ISS score	Shock	Shock, Sepsis	Not mentioned
Lai WH et al. Scand J Trauma Resusc Emerg Med. 2016;24(1):136.[16]	Retrospective study (1/1/2009 – 31/12/2014)	All trauma patients admitted in hospital (Taiwan trauma registry)	78/14504 (0,54%) general in trauma 45/2789 (2.1%) ICU trauma patients	KDIGO 2012	Within 24 hours	3/78 (3.8%) RRT	Not mentioned	Age, Diabetes, Hypertension, Coronary artery disease	ISS score, Shock, GCS<8, Longer transport time	Shock, Intracerebral hemorrhage,	Shock	Not mentioned
Haines RW et al, Sci Rep. 2018;8(1):3665. [28]	Single-center retrospective observational study (2/2012 – 31/10/2014)	Only trauma ICU patients	163/830 (19.6%)	KDIGO 2012	Within 7 days (median time 2.7 days)	42/830 (5.1%)	53/163 (32.5%) vs 103/667 (15.4%)	Age, Charlson score	NISS score, ISS score, Abdominal or pelvic trauma, ER SBP, ER lactate, PRBC’s transfused in the first 24 h, first ALT, CK, SCr, and phosphate	First Cr, Phosphate, Blood transfusion	SAPS II score, Blood transfusion	Not mentioned
Harrois A et al, Crit Care 2018;22(1):344. [13]	Prospective observational multicenter study (5/2011-6/2014)	3 French level-1 trauma centers	13% total AKI R: 7% I:3.7% F:2.3%	RIFLE	96% within 5 days	1.6% RRT	Twofold increase ICU mortality	Not the age, SAPS II score	ABP, Maximum heart rate, ISS score	Renal trauma, Lactate, Hemorrhagic shock, RBC transfusion	SAPS II score, SOFA score, CPK peak	Not mentioned
Perkins ZB et al, PLoS One 2019;25: 14(1):e0211001. [8]	Single-center prospective observational study (1/2007-31/2016)	From ER admission-ICU-Hospital discharge	178/1410 12.6% KDIGO stage 1: 66.3% 2: 10.1% 3: 23.6%	KDIGO 2012	2 (1–5) days	38/178 (21.4%)	47/178 (26.4% in AKI patients) 128/1232 (10.4% in non-AKI)	Age, Diabetes	ISS score, Blunt injury, Shock	Volume overload, Blood transfusion, Admission SBP, Lactate	Volume overload, Blood transfusion, Vasopressors, Nephrotoxic drugs	Not mentioned
Leditzke K et al, In Vivo. 2021;35(5):2755–62.[19]	Single-center retrospective observational study (10/2016-01/2018)	ICU trauma patients with ISS>16, admitted within 6 hours after injury	18/39	NGAL, Serum creatine, Serum urea	1.2±1.4 days (range:0–5 days)	Not mentioned	18% mortality	CKD	Severe injury (ISS>16)	Catecholamines	MV, Catecholamines, Sepsis, NGAL>177 ng/ml	Not mentioned
Yasuda R et al, Front Med (Lausanne). 2024 Feb 23;11:1346183.[20]	Single-center prospective observational study (10/2019-02/2020)	ICU trauma patients	15/100	LFAB	6–12 hours after trauma	Not included	Not mentioned	Not mentioned	ISS	Contrast media, shock	Shock	Not mentioned
Martinez et al, Crit Care 2024;28(1):382.[29]	Multicenter retrospective cohort study (French Traumabase registry) (1/1/2012 – 1/6/2023)	ICU trauma patients, CK>5000 U/L	1544/8592 severe RM		Not mentioned	Not mentioned	4% increase 30-day mortality	Not mentioned	ISS score	Not mentioned	Not mentioned	Not mentioned

Abbreviations: ABP: Arterial Blood Pressure, AIS: Abbreviated Injury Scale, AKI: Acute Kidney Injury, APACHE II score: Acute Physiologic Assessment and Chronic Health Evaluation II score, BE: Base excess, BMI: Body Mass Index, CKD: Chronic Kidney Disease, ER: emergency room, GCS: Glasgow Coma Scale, ISS: Injury Severity Score, MV: Mechanical Ventilation, NISS: New Injury Severity Score, OR: operating theatre, PRBC: packs of red blood cell, RM: Rhabdomyolysis, SAPS II score: Simplified Acute Physiology II score, SBP: Systolic Blood Pressure, SOFA score: Sequential Organ Failure Assessment score

### Risk factors of TrAKI emerged in different time points of trauma treatment

#### 1. Demographic factors and comorbidities

Demographic characteristics and previous medical history are distinguished contributors to critical illness’s outcome (Table 2). Besides, advancing age and comorbidities, such as diabetes, hypertension and coronary disease, is known to be associated to subclinical renal dysfunction or coexisted nephropathy [8,25] and higher AKI incidents upon critical illness [16]. In studies including more comparable and younger population, the age didn’t aggravate the AKI rate [10,23,26,27] (Table 1).

**Table 2.** Risk factors of TrAKI in different time points

	**Risk factors**	**Intervention**	**Pathophysiologic mechanism of TrAKI**	**Clinical and/or biochemical markers**
Physical characteristics	Older age, Male gender, African American, Obesity			BMI
Comorbidities	Chronic Kidney Disease, Diabetes mellitus, Chronic hypertension, Chronic heart failure, Cirrhosis, Chronic obstructive pulmonary disease, Hematologic malignancy	Antiplatelet drugs,		APACHE II, III score SAPS II score, Charlson score
At hospital admission	Abdominal trauma, Pelvic trauma, Blunt and penetrating trauma, Renal trauma, Brain Injury		Hemorrhagic shock, Hypovolemia, Hypoxia, Renal hypoperfusion	Transportation time, ISS, NISS AIS, Admission lactate value, Minimum prehospital MAP, Maximal prehospital HR, Duration until trauma center admission, GCS
First 12-24 h (ER and OR treatment)	Uncontrolled hemorrhage		Renal hypoperfusion, Renal hypoxia	Coagulopathy, Hypoxemia, Hypothermia, Lactemia, Viscoelastic assays, Acidosis
	Resuscitation process	Number of transfused units of PRBC, Fluid overload, Need for vasoconstrictives	I/R, Oxidative stress, Systematic inflammatory response	
	Diagnostic process	Intravenous contrast agents	Renal toxicity	
	Damage control surgery	Emergency surgery, ACS, Fluid overload, Massive transfusion	Renal hypoperfusion, Renal hypoxia, RM, Systematic inflammatory response	
ICU admission (first 5 days – weeks)	Illness severity			SAPS II score, APACHE II or III score, SOFA score
	ICU treatment	Mechanical ventilation	Systemic inflammatory response, Worsening IAH	Hypoxemia, Hypothermia, Lactemia, Acidosis, Coagulopathy, Viscoelastic assays
		Vasoactive therapy (noradrenaline, vasopressin)	Renal hypoperfusion	
		Fluid infusion	I/R, Oxidative stress	
		Blood transfusion		
		Nephrotoxic drugs (diuretics, non-steroidal anti-inflammatory drugs, aminoglycosides, glycopeptides, contrast media)	Renal toxicity	
	ICU complications	Fluid overload	Systemic inflammatory response, Worsening IAH	CK, Myoglobin, Urea, Creatinine, Diuresis, Acidosis, Lactemia, Electrolytic abnormalities, IAP values, AKI biomarkers (NGAL, L-FABP, IGFBP-7 and TIMP-2 etc)
		RM	Intrarenal vasoconstriction, Ischemic injury, Tubular obstruction, Oxidative injury, Renal inflammation	
		ACS	Renal hypoperfusion	
		Sepsis	Renal hypoperfusion, Renal oxidative injury	
		Multiorgan dysfunction		

Abbreviations: AIS: Abbreviated Injury Scale, ACS: Abdominal Compartment Syndrome, AKI: Acute Kidney Injury, APACHE II score: Acute Physiologic Assessment and Chronic Health Evaluation II score, BMI: Body Mass Index, CK: Creatine Phosphokinase, ER: emergency room, GCS: Glasgow Coma Scale, HR: Heart Rate, IAH: Intra-abdominal Hypertension, IAP: Intra-abdominal Pressure, IGFBP-7: Insulin like growth factor binding protein-7, ISS: Injury Severity Score, L-FABP: Liver fatty acid-binding protein MAP: Mean Arterial Pressure, NISS: New Injury Severity Score, NGAL: Neutrophil Gelatinase-Associated Lipocalin, OR: operating theatre, PRBC: packs of red blood cell, RM: Rhabdomyolysis, SAPS II score: Simplified Acute Physiology II score, SOFA score: Sequential Organ Failure Assessment score, TIMP-2: Tissue inhibitor of metalloproteinases 2.

Moreover, there are studies were obesity predisposed AKI in trauma patients [11]. A pathophysiologic contribution of adiposity to AKI is logical, since excess adipose tissue results in a chronic systemic inflammation, linking to AKI [30], while obesity has known associations with chronic glomerulopathy [30,31]. However, it is not clear yet, whether the BMI-AKI (Body Mass Index) association reflects a pathogenetic role of obesity or an artifact driven by the method of defining AKI, considering the existed definition limitations, using only KDIGO criteria [32], in obese patients [11].

#### 2. Pre-hospital related AKI factors (from the time of injury to hospital arrival)

This period includes the first hit, i.e. the major trauma itself, minimal or no effort to stabilize under prehospital conditions and transferred time to trauma center (Figure 1) (Table 2). It can last a few minutes to several hours [23,26,27]. Factors that contributed to AKI are markers of hypoperfusion, hypoxia and metabolic aggression upon ER arrival, such as admission lactate value, hemorrhagic shock, minimum prehospital mean arterial pressure (MAP) and maximal prehospital heart rate (HR), injury severity (ISS), renal trauma, abdominal or pelvic trauma and delayed hospital admission [13]. The main AKI pathophysiologic mechanism during this period is renal hypoperfusion, hypoxia, hypothermia and acidosis [33].

Although the kidneys receive an arterial blood supply that highly exceeds the oxygenation needs of the renal tissue, there is an intra-renal system of arterio-venous (AV) oxygen shunting that results in renal vein having higher partial oxygen pressure than intra-renal microvasculature, thus protecting renal tissue from hyperoxia-associated injury [34]. However, this AV-shunting may put kidney oxygen delivery at risk of renal hypoperfusion [10].

The transfer time from trauma scene to ER seems to be related with the possibility of renal impairment [23,26,27]. Patients directly admitted to a trauma center were less likely to experience TrAKI, than those who were secondarily transferred to the appropriate hospital, due to earlier hemorrhage control and hemodynamic stabilization [1,9,13,27] (Table 1).

Elevated initial lactate, as an index of tissue hypoxia, has also been correlated with the development of AKI in several cohorts [9,10,23,28]. Its value upon ER arrival indicates a cumulated metabolic debt due to tissue hypoperfusion and underresuscitation, all of which can trigger AKI. Moreover, kidney contributes to the removal of up to 25–30% of lactate load, mostly through its metabolism rather than excretion, especially by acidosis, where kidney’s ability to remove lactate is increased. Consequently, renal hypoperfusion could impair lactate removal [35].

Apparently, minimizing the severity and duration of kidney hypoperfusion and hypoxia, in prehospital conditions, could be beneficial to prevent TrAKI. Moreover, hemodynamic parameters, such as minimum prehospital MAP or maximum HR [8,13], and biomarkers indicating hypoperfusion and tissue hypoxia, such as lactate [8,13,23], Neutrophil Gelatinase-Associated Lipocalin (NGAL) [19] or Liver fatty acid-binding protein (L-FABP) [20] during transportation and upon ER admission, could have predictive value.

#### 3. Pre-ICU related AKI factors

This period could last a few minutes to several hours and includes the second hit, due to continual hemorrhage, resuscitation process, diagnostic procedures, and major surgical operations (Figure 1) (Table 2). Various stabilizing interventions that ensure oxygen transport to body cell can be lifesaving, while might also promote TrAKI, which manifests within the first post-traumatic 72 hours (Figure 2). The main pathophysiologic mechanism during this period except prolonged renal hypoperfusion and hypoxia, due to uncontrolled shock, seems to be ischemia-reperfusion injury due to aggressive resuscitation, nephrotoxicity and systemic inflammation response due to antibiotics or radiographic contrast agents and emergent surgery or the major trauma itself, respectively [33].

##### Severity of trauma injury

The severity of trauma injury has been associated with AKI [8,9,11,13,16,23,24,25,26,27,28,29] (Table 1). Injury severity score (ISS), as an established grading score, has been correlated with morbidity and mortality [1,9,10,36,37]. To be mentioned that the basic limitation is that ISS appraises only injuries and not their physiological consequences [1,9,10,36,37]. For example, a patient with severe brain injury and spinal cord trauma has a high ISS but not necessarily hemorrhagic shock or severe systemic inflammatory syndrome, that could both lead to organ dysfunction. On the other hand, a patient with long bone fractures, although has a low ISS, is likely to need massive transfusion and multiple surgical interventions, leading to severe RM and inflammatory response. However, even though ISS may not be an accurate AKI risk factor, it has been related to renal dysfunction [8,11,16,18,23,24,25,26,27,28,29].

Furthermore, direct renal trauma has also been associated with higher AKI prevalence, since kidney parenchyma and vessel injury reflect functional nephron loss, leading to glomerular filtration inability [12,13]. Abdominal injury and increased abbreviated injury scale (AIS), have also reported as risk factors for AKI, since they may indicate the severity of retroperitoneal or intraperitoneal hemorrhage, leading to hypovolemia, renal hypoperfusion and possible ACS [1,11,12,13]. Besides, damage control surgery packing in such cases could further deteriorate the existed intra-abdominal hypertension (IAH), worsening [17,38].

##### Brain injury severity

There is increasing evidence for the association between TrAKI and the severity of brain injury [9,12,14,15,18,24], since a low Glasgow Coma Scale (GCS) score has been related to hypoventilation, hypoxemia and secondly to necessity of critical care treatment [17] (Table 1). Moreover, routinely treatment with mannitol in intracranial hypertension has been associated with renal deficiency, while adequate intravascular volume maintenance and low-dose (0.3 g/kg) mannitol infusion, intracranial pressure (ICP)-directed, seems to be beneficial on the occurrence of AKI [15]. The use of antihypertensive agents or an aggressive systolic blood pressure (SBP) reduction in patients with intracerebral hemorrhage have, also, been associated to trigger AKI [9]. Furthermore, severe traumatic brain injury patients, that usually need prolonged ventilatory support, ICU and hospital stay, are exposed to a great number of delayed complications, triggering “late” AKI [24].

##### Hemorrhagic shock

Traumatic hemorrhagic shock is a state of inadequate cellular energy production and a major cause of trauma mortality and serious complications among survivors, such as AKI [39]. Acute, severe hemorrhage, by decreasing intravascular blood volume, can cause hypovolemia, hemodynamic instability, cardiac output reduction, inadequate tissue perfusion, cellular hypoxia, organ dysfunction and ultimately death [40].

Consequently, it can affect kidney homeostasis minimizing oxygen delivery through multiple mechanisms. Firstly, hypotension and reduced cardiac output due to hypovolemia directly decreases renal arterial blood supply, when arterial pressure level reaches the inferior limit of autoregulation [37]. Further, the recruitment of mechanisms aiming at stabilizing circulation, such as the activation of the sympathetic system and the renin/angiotensin axis leads to intra-renal vasoconstriction, particular at the efferent limb creating post-glomerular tubular blood hypoperfusion [41]. This is, additionally, aggravated by post-hemorrhagic anemia, which decreases the absolute amount of oxygen delivered to tissues [42]. Indeed, histological studies of hemorrhage-induced AKI demonstrated evidence of acute tubular necrosis, such as loss of tubular brush border, epithelial cell vacuolation up to epithelial desquamation, while hypoxia-inducible factor-1a (HIF-1a) is detected early [42].

On the other side, while hemorrhage itself seems to induce inflammation, resuscitation leads to renal oxidative stress, since the amount of produced superoxide anion can trigger cell apoptosis [37]. Experimental evidence show that 90 minutes of hemorrhagic shock are enough to raise the renal expression of Tumor Necrosis Factor-a (TNFa) and interleukin-6 (IL-6). Adhesion molecules, such as, intercellular adhesion molecule-1 (ICAM-1) and E-selectin are upregulated in endothelial cells, promoting leukocyte deceleration and transmigration into the renal parenchyma [43]. Interestingly, mere ischemia is not capable to induce those alterations, since reperfusion is essential for them to fully develop. Other potent pro-inflammatory mediators, like high-mobility-group-box 1 (HMGB-1) or the receptor for advanced glycation endproducts (RAGE) have also been reported to rise in the renal tissue following hemorrhage, while the role of mediators released from the ischemic gut is also to be taken into account [44].

##### Fluid resuscitation

Severe hemorrhage treatment includes primarily the blood lose minimize, and at the same time monitoring-guided fluid infusion and coagulopathy management, aiming to restore intravascular volume, hemodynamic stability and tissue perfusion [33,39]. Therefore, fluid resuscitation is a cornerstone of pre- and in-hospital trauma care [40]. However, aggressive fluid resuscitation has been associated with exacerbating acute bleeding by increasing hydrostatic pressure and decreasing clot firmness [40].

Permissive hypotension based on maintaining blood pressure at a lower-than-normal level, that ensure an adequate organ perfusion, has been reported to reduce bleeding, minimize blood transfusion and improve mortality [45,46]. Current recommendations suggest for traumatic hemorrhagic shock without severe head injury, to titrate fluids and use early vasopressors to maintain permissive hypotension (target SAP 80–90 mm Hg or MAP 60–65 mm Hg), until hemorrhage control [47]. If traumatic brain injury is associated to severe hemorrhage, guidelines recommend targeting a SBP above 110 mm Hg [47].

Hypotensive resuscitation, in traumatic hemorrhagic shock, aims to minimize the resuscitation volume and blood product transfusion avoiding further bleeding and sustaining organ perfusion, since surgical treatment [45,46,48]. Although hypotensive resuscitation has been correlated with better outcome and non-significant AKI incidence [45,46,48], no study indicates the optimal level of MAP in hemorrhagic shock, in order to prevent renal disfunction [37]. Therefore, a short-term hypotension could be acceptably tolerated, while in case of patient’s deterioration, adequate fluid volume should be infused [49]. To be mentioned that retrospective data indicate an association between prolonged permissive hypotension and organ hypoperfusion and multiorgan dysfunction [50], while seems to increase mortality among elderly trauma patients [51,52,53].

Fluid administration could be harmful itself, due to either the large infused volume or the fluid composition. Fluid resuscitation should be monitoring-guided [15], since while hypovolemia could cause renal ischemia, fluid overload may lead to severe tissue and pulmonary edema, cardiac dysfunction, gastroenteric dysmotility, coagulation disorders, ACS and immunological disturbance [39]. Besides, volume overload has been associated to reperfusion-related inflammation, as it has been shown in experimental models where extended edema, capillary leakage and leukocyte adhesion were observed [39]. In addition, it has been demonstrated that a controlled infusion of crystalloids in elderly patients was associated with a better outcome [54], while when trauma patients received more than 1.5 liter of crystalloid in the ER a higher mortality was marked [55]. Jones et al, also, reported proportional higher mortality and prolonged need of mechanical ventilation, when administrated more than 5 liters crystalloids in patients within 24 hours of trauma [39].

Current guidelines advocate for goal directed fluid administration with repeated 250 ml fluid boluses until regaining of specific blood pressure levels, before bleeding control [40]. These strategies, however, have not been evaluated by large prospective randomized clinical trials, and demographic factors such as age, comorbidities and previous medical history could modify the resuscitation strategy [40]. Recent data, for example, suggests that the elderly could benefit from higher blood pressure goals [45,51,52,53]. Whether larger volumes of infused fluids and blood products or earlier vasopressor initiation would be the appropriate tool to obtain these targets remain unknown [40].

The type of infused fluids seems to play a crucial role in kidney hemostasis and final outcome. Isotonic crystalloid solutions are the first-line therapy for fluid expansion, to correct post-traumatic hypovolemia [37,40]. Normal saline (NaCl 0.9%) was considered as the basic resuscitation fluid until recently, since Ringer’s Lactate, with o slightly lower osmolarity (308 vs. 273 mmol/l respectively) was supposed to increase intracellular space volume and worsening intracranial hypertension [37]. However, NaCl 0.9% has been related to hyperchloremic acidosis, more renal adverse effects [56] and higher mortality compared to Ringer’s Lactate solution [57]. On the other hand, synthetic colloids were proposed as potential fluids with great oncotic ability [37]. However, since June 2013 [58] the US Food and Drug Administration withdrawn hydroxyethyl starch, due to the increase incidence of AKI and mortality, especially among septic patients [33]. On the other hand, although hypertonic saline (7.5% saline) has long been considered to have potential benefits due to rapid restoration of intravascular volume with small intravascular volume administration, reduction of intracranial pressure and modulation of inflammatory response, failed to demonstrate an outcome benefit [59]. After these, although the SPLIT [60], BaSICS [61] and PLUS trial [62] failed to show a benefit regarding mortality or AKI incidence, using balanced crystalloids, like Plasmalyte, as resuscitation fluid, compared with Normal Salin, balanced crystalloids have been promoted as more nephroprotective [63].

##### Blood transfusion

Previous studies have presented an increased risk of AKI among trauma patients that required massive blood products transfusion [8,10,11,13,16,23,26,28] (Table 1). However, it is not clarified if the necessity for the transfusion or the transfusion itself is related to AKI. The amount of transfusion could be an index of trauma severity, the blood volume loss, hypovolemia, tissue hypoperfusion and finally tissue ischemia. Besides, transfusion may provoke systemic inflammatory response and cytokine release, leading to renal impairment [11,13]. Additionally, stored red blood cells, following transfusion, gradually release hemoglobin, which not only has direct toxic effects on renal tubules, but also depletes nitric oxide (NO), impeding its vasodilatory effects upon renal vessels [64]. In conclusion, current guidelines recommend minimizing the need of transfusion. After early hemorrhage control, the treatment should focus on correction of metabolic derangements and hypothermia to decrease coagulopathy and substitution according to viscoelastic assays [65] and blood gases analysis with tranexamic acid, fibrinogen concentrate, red blood cells, plasma and platelets [66].

##### Nephrotoxic agents

Computed tomography routinely evaluates the severity and anatomical distribution of traumatic area. In most cases, the use of potential nephrotoxic contrast media is required [25,37]. In addition, other possible renal harmful agents, such as aminoglycosides and non-steroidal-anti-inflammatory drugs are widely dispensed to trauma patients. Although latest contrast mediators supposed to be less nephrotoxic, thoughtful awareness is needed regarding to the necessity for contrast assisted imaging, minimizing non-essential radiological exams and unnecessary probable detrimental antibiotics [37].

#### 4. ICU related AKI factors

Multiorgan, long-lasting ICU support of trauma patient and possible critical illness’ complications act as a third hit (Figure 1). Both patient-derived and trauma-specific AKI risk factors have been identified (Table 2). This period could last from a couple of days to several weeks or even months and several pathophysiological mechanisms, such as RM, ischemia-reperfusion injury, IAH, systemic inflammation response, renal toxicity or even sepsis, promote “late” TrAKI, that manifests within days to weeks after traumatic injury (Figure 2).

##### Rhabdomyolysis

Rhabdomyolysis severity, as assessed by the creatinine kinase (CK) peak, has been found as an additional independent risk factor for AKI [13,23,25,26,29] (Table 1). RM can be induced by direct insult, such as crush syndrome, extensive muscular injury or major surgery, and indirect muscular injury due to massive hemorrhage or vascular damage [29]. It can be presented either as asymptomatic serum myoglobin and CK elevation, or even as severe AKI with serious disseminated intravascular coagulation. CK, as an index of RM severity, is related to the amount of possible, nephrotoxic, intramuscular content release and reaches the highest level within 17 hours post-traumatic [26]. RM-related AKI is caused by renal vasoconstriction due to myoglobin, tubular obstruction, tissue ischemia, oxidative stress and inflammation [26,37]. Myoglobin promotes superoxide anion production in the vessel wall of afferent arterioles, reducing NO bioavailability. As a result, myoglobin enhances Angiotensin II-induced constriction of renal afferent arterioles, causing renal hypoperfusion and tubular hypoxia [67]. Moreover, myoglobin, may accumulate in renal tubules, which is exacerbated in acidosis, caused by hypovolemia, obstructing renal tubules [37]. Finally, it activates endothelium and neutrophils leading to renal inflammation and ultimate renal fibrosis [37].

The diagnosis of RM is likely to be underestimated among trauma patients. Both CK and myoglobin levels are commonly used biomarkers for diagnosing RM [29]. Implementing measures to diagnose RM and assess its severity can help evaluate patient prognosis from the early days of treatment. Currently, there is no specific therapy, except from preventive measures to mitigate renal impairment [68]. Such measures include primary volume resuscitation, in order to promote renal tubule flow, dilute nephrotoxins such as myoglobin and supply adequate renal perfusion to prevent AKI [68]. To be mentioned that administration of sodium bicarbonate and diuretics, for prevention of AKI, are not recommended [69]. Secondly, electrolytic abnormalities, such as hyperkalemia, hyperphosphatemia, hypocalcemia and hypomagnesemia, should be corrected [70]. Finally, the utilization of RRT in patients with RM should be based on AKI indications since it has no effective preventive role [69,70,71].

##### Abdominal hypertension

Traumatic IAH, defined as an elevation of intraabdominal pressure (IAP) above 12 mm Hg, and ACS as a sustained IAP above 20 mm Hg [37], has been reported to constitute a crucial cause of renal impairment due to kidney vessels and parenchyma compression and cardiac output reduction [72,73]. Major intrabdominal and/or retroperitoneal bleeding, due to pelvic or abdominal trauma could cause IAH in 0–37% of incidence, raising AKI prevalence in 42–50% [72]. AKI in ACS seems to be related to the external renal compression and consequential hypoperfusion. Experimental studies reported direct compression of renal veins and affected GFR, which were returned back to normal as soon as vein flow was restored. This simulates abdominal surgical decompression, which restores diuresis in ACS [73]. Volume overload and invasive mechanical ventilation, in such cases, could further deteriorate renal interstitial edema, increasing intrarenal pressure rapidly, due to capsula’s low compliance [73,74].

Transvesical intra-abdominal pressure measurement remains the strategy of choice to diagnose IAH and ACS [75]. Biomarkers, such as urinary and plasma intestinal fatty acid binding protein (iFABP), have not proved value in predicting IAH-related complications in ICU patients [76,77]. Damage control surgery (DCS) is a surgical strategy that was primarily used in unstable trauma patients and includes an abbreviated surgical procedure aimed at control hemorrhage, open abdomen treatment and delayed repair of anatomical injuries [75]. Of note, deepening of sedation in mechanical ventilated trauma patients with IAH, had not a significant effect in IAH controlling, while required increased vasopressor doses, which finally decrease abdominal perfusion pressure [78].

## Conclusion

Trauma-related AKI turns out to be a common complication among severe injured critical patients. Although the subgroup of trauma patients is likely younger, with less comorbidities, as compared with other critically ill patients, TrAKI further deteriorates the outcome. The biphasic pattern of incidences of TrAKI reflects the different underlying pathophysiology, highlighting unique risk factors at different timepoints of trauma treatment and probable distinct prognostic implications, regarding different outcomes, such as AKI recovery, CKD development, morbidity, mortality and health-care cost expansion. Earlier recognition of patients in a high risk of TrAKI, might create a window of opportunity to prevent further kidney damage.

The majority of existing studies are retrospective cohorts, based on national trauma registries, including small samples and reporting considerable heterogeneous outcome conclusions. These surveys are extended for long time periods, over which the management of trauma may have evolved (e.g. withdrawal of synthetic colloids, availability of RRT) and rarely present data on renal recovery, CKD incidences or long-term RRT requirement. Demographic, clinical and biochemical parameters related to trauma treatment could constitute a valuable predictive model to detect patients in a high risk to develop TrAKI and need RRT. The combination with diagnostic biomarkers may improve the accuracy and sensitivity of such tools, promoting novel approaches for prevention and treatment of TrAKI, increasing the power and homogeneity of future clinical trials.
